# Case Report of Complete Radiological Response of a Thalamic Glioblastoma After Treatment With Proton Therapy Followed by Temozolomide and Tumor-Treating Fields

**DOI:** 10.3389/fonc.2020.00477

**Published:** 2020-04-21

**Authors:** Marco Stein, Hildegard Dohmen, Bernhard Wölk, Fabian Eberle, Malgorzata Kolodziej, Till Acker, Eberhard Uhl, Alexandra Jensen

**Affiliations:** ^1^Department of Neurosurgery, Justus-Liebig University, Giessen, Germany; ^2^Institute of Neuropathology, Justus-Liebig University, Giessen, Germany; ^3^Department of Neuroradiology, Justus-Liebig University, Giessen, Germany; ^4^Department of Radiotherapy and Radiooncology, UKGM Marburg, Marburg, Germany; ^5^Marburg Particle Therapy Center (MIT), Marburg, Germany; ^6^Department of Radiation Oncology, UKGM Giessen, Giessen, Germany

**Keywords:** glioblastoma, TTFields, radiological response, proton therapy, biopsy

## Abstract

Glioblastoma (GBM) is the most common and aggressive primary brain tumor in adults. We present a case of a 42-year-old male patient presenting with headache and vomiting. Imaging demonstrated obstructive hydrocephalus and a ring-enhancing lesion in the right posterior thalamus. After endoscopic third ventriculostomy and stereotactic biopsy, the histopathologic diagnosis of a malignant glioma was confirmed by DNA methylation array as GBM isocitrate dehydrogenase wild type. The patient was treated with combined treatment of chemoradiation with temozolomide (TMZ) including proton boost, TMZ maintenance, and tumor-treating fields. In this case report, complete radiological response was observed 1 year after the end of radiation therapy.

## Introduction

Glioblastoma (GBM) is the most common and malignant primary brain tumor in adults with poor outcomes and limited treatment options in isocitrate dehydrogenase (IDH) wild-type GBM. Current therapy consists of maximal safe surgical resection, photon radiotherapy, chemotherapy, and, according to the National Comprehensive Cancer Network Guidelines (1), the addition of tumor-treating fields (TTFields) therapy. Despite intensive research over the past years, outcomes of patients with GBM remain poor.

Tumor-treating fields therapy combined with maintenance temozolomide (TMZ) chemotherapy significantly increases overall survival (OS) and progression-free survival in primary GBM (2). Combined proton and photon radiotherapy could be beneficial in terms of risk reduction for treatment-related adverse effects (3).

Currently, there have been no reports on the combination therapy of TTFields with proton radiotherapy in primary GBM. Complete radiological response after subtotal resection, chemoradiation, and TTFields is reported (4). In this report, we present a case of a patient with biopsied thalamic GBM IDH wild-type showing a complete radiological response after chemoradiation with TMZ, proton boost therapy, and TMZ maintenance in combination with TTFields.

## Clinical Details and Treatment Modalities

**Figure 1:**
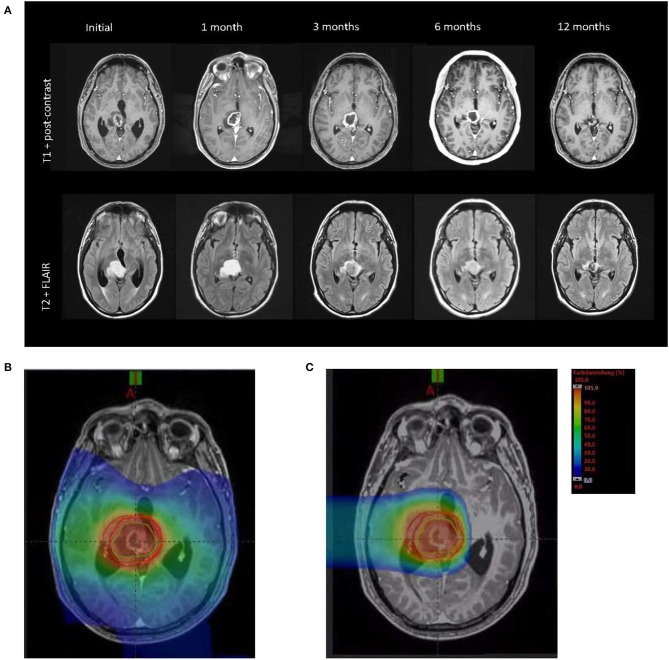
**(A)** MRI (T1 postcontrast and T2-FLAIR) showed pseudoprogression at 1 month after radiation therapy and complete radiological response after 1 year. **(B)** Radiation field photon therapy (25 × 2 Gy). **(C)** Radiation field proton therapy (5 × 2 Gy RBE).

**Figure 2:**
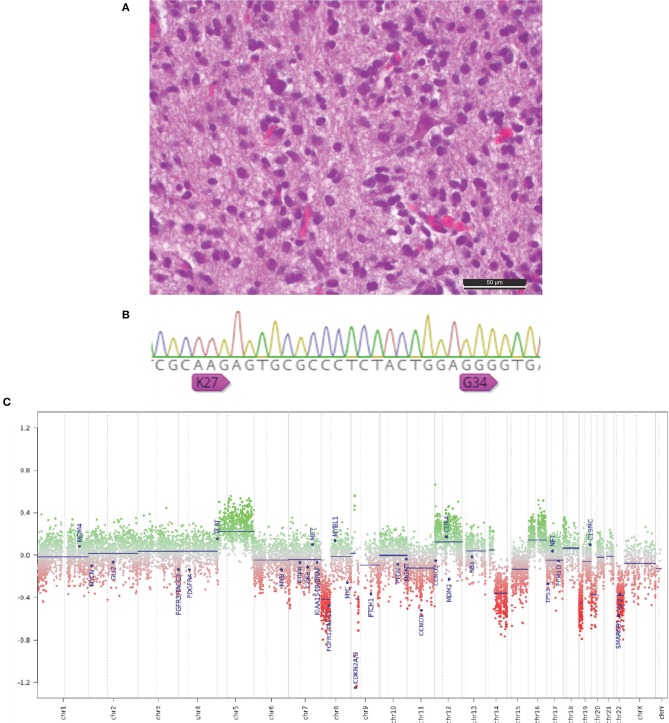
**(A)** Hematoxylin and eosin–stained section of an anaplastic pleomorphic diffuse infiltrating astrocytic brain tumor (scale bar, 50 μm). **(B)** Sanger sequencing with evidence of wild-type H3F3A (K27, G34). **(C)** Representative copy number profile plots showing several chromosomal amplifications and deletions, especially CDKN2A/B deletion.

A 42-year-old right-handed man presented with headache and vomiting for 1 week in July 2017. The family and medical history of the patient was unremarkable for oncology or neuro-oncology diseases. Brain magnetic resonance imaging (MRI) demonstrated obstructive hydrocephalus and a ring-enhancing lesion in the right posterior thalamus (Figure 1A). Endoscopic third ventriculostomy and stereotactic biopsy of the lesion were performed. Postoperatively, the patient was clinically stable with a Karnofsky Performance Status (KPS) score of 90. Histopathologic examination showed endothelial proliferation and areas of necrosis and resulted in the diagnosis of a GBM [World Health Organization (WHO) grade IV] IDH wild type, H3F3A (K27, G34) HIST1H3B/C and H2H3C wild type, TERT promoter wild type (C228 and C250), KIAA1549-BRAF wild type, and unmethylated MGMT promoter (Figure 2). We tried using brain tumor methylation classifier, but no matching methylation class was found.

**Figure 3:**
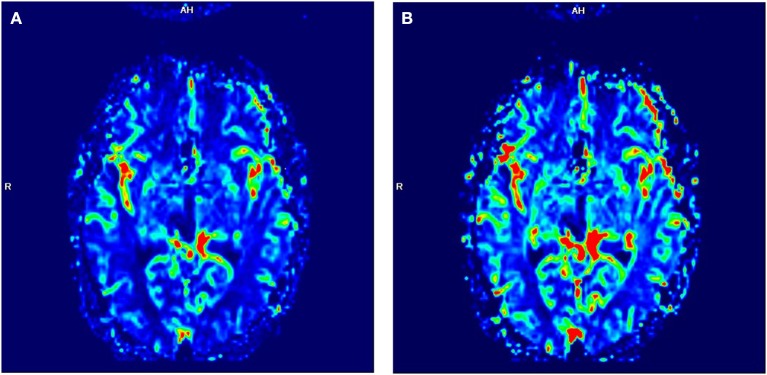
Perfusion-MRI. Neither increased CBF **(A)** nor increased CBV **(B)** was detectable in the former tumor area 18 months after the end of radiation therapy. In the color-coded pictures, red indicates high blood flow (BF); green indicates moderate BF, and blue indicates low BF.

In view of the tumor site, resection was not feasible. Consequently, the patient received chemoradiation therapy. Radiation therapy was performed with 50.0-Gy photons in 2.0-Gy fractions, followed by a proton boost with 10-Gy equivalent [Gy (RBE)] in 2.0-Gy (RBE) fractions. Tumor-treating fields therapy in combination with TMZ was initiated 4 weeks after completion of chemoradiation; TMZ maintenance was completed per protocol after six cycles. The first MRI 4 weeks after the end of radiation therapy showed a tumor increase in T1 and T2 fluid-attenuated inversion recovery (FLAIR). However, the patient remained clinically stable with a KPS of 90, suggesting that most likely a pseudoprogression of the tumor was present. Corticosteroids were used only in the first week after admission and during the radiation therapy. Tumor-treating fields therapy was continued, and in due course, the tumor decreased on serial MRI scans. One year after radiation therapy, a complete radiological response was observed (Figure 1A). Tumor-treating fields therapy was continued from the beginning of TMZ maintenance until June 2019. Overall, the patient received TTFields therapy for 20 months. The TTFields usage (rate of compliance), that is, the duration of TTFields treatment per month, was 84%. This is well above the recommended treatment duration threshold of 75% and supports the feasibility of combining TTFields with proton therapy (5). The current follow-up time is 27 months after initial diagnosis, and the patient still shows radiological complete response. Furthermore, a contrast-enhanced perfusion MRI in February 2020 showed reduced cerebral blood flow (CBF) and cerebral blood volume (CBV) in the former tumor area, compared to the contralateral brain hemisphere (Figure 3). On clinical examination, the patient showed no neurological deficits, and in November 2019, the patient is still stable.

## Methods

The tissue specimen was histopathologically classified according to the current WHO classification (6). Areas with the highest tumor cell content (≥70%) were selected for DNA extraction. DNA was extracted from formalin-fixed paraffin-embedded tissue using the automated Maxwell system (Promega, Fitchburg, MA, USA) according to the manufacturer's instructions. DNA concentration was quantified using the Qubit ds DNA HS Assay kit (Invitrogen, Carlsbad, CA, USA). Approximately 200–500 ng of DNA was used as input for DNA methylation analysis. DNA was treated with sodium bisulfite. For the analysis, the Infinium® MethylationEPIC BeadChip (850 k) (Illumina, Carlsbad, CA, USA) and Brain Tumor Classifier (http://molecularneuropathology.org) was used to determine the DNA methylation status of more than 850,000 CpG sites, respectively, following the manufacturer's guidelines. The genome-wide DNA methylation profile was compared with more than 2,800 tumors in a reference library. The classifier score (between 0 and 1) expresses the similarity with a reference group (7). For a prediction, a methylation score with a cutoff of 0.9 or 0.5 is required. Irrespective of this, a copy number profile was calculated.

### Molecular Analysis

#### O(6)-Methylguanine-DNA Methyltransferase (MGMT) Promoter Methylation

After extraction of DNA from the paraffin material of the tumor, DNA was treated with sodium bisulfite, and methylation-specific polymerase chain reaction (PCR) analysis to detect MGMT promoter methylation was performed.

#### Telomerase Reverse Transcriptase Promoter Mutation and IDH1/2 Mutation

After DNA extraction, amplifications of the telomerase reverse transcriptase (TERT) promoter (target region: chr5: 1,295,189-1,295,379; GRCh37/hg19) and the IDH1 and IDH2 genes by PCR were investigated. Sanger sequencing of the TERT promoter (hotspot mutation sites NC_000005.9: g.1295228G and NC_000005.9: g.1295250G; GRCh37/hg19) and the IDH1 and IDH2 gene (codon 132 and codon 172) was performed.

##### KIAA1549-BRAF fusion transcript

RNA was extracted from the paraffin material of the tumor, and cDNA was synthesized. Amplifications of KIAA1549: BRAF fusion transcripts were observed by PCR (type A: IAA1549 Ex1-16: BRAF Ex9-18; type B: KIAA1549 Ex1-16: BRAF Ex11-18; type C: KIAA1549 Ex1-15: BRAF Ex9-18).

##### H3 histone analysis

After extraction of DNA from the paraffin material amplifications of the H3F3A (histone 3.3) gene, HIST1H3B, HIST1H3C (histone 3.1), and HIST2H3C (histone 3.2) genes were investigated by PCR. Sanger sequencing of the H3F3A (codon 27 and codon 34) gene and of HIST1H3B/C, and HIST2H3C genes (codon 27) were performed.

### Proton and Photon Radiation Therapy

Based on initial experience in mixed-beam radiotherapy with protons, the patient received 25 × 2-Gy photon radiotherapy followed by 5 × 2-Gy RBE protons. After immobilization in a bespoke thermoplastic head mask (ITV®), treatment planning was carried out based on a native computed tomography (CT) scan with 3-mm slice thickness both for photon and proton radiotherapy.

#### Photon Radiotherapy

Planning target volume definition for photon radiotherapy (PTVphotons) was based on international guidelines (8) following image coregistration of axial T2 FLAIR and T1 fat-saturated contrast-enhanced MRI scans. Treatment planning for intensity-modulated radiotherapy in rotation technique (RapidArc^©^) including optimization and dose calculation was carried out with the Varian Eclipse® treatment planning system. Fifty gray was prescribed to the median of PTVphotons requiring encompassing the volume with at least 95% prescription isodose. RapidArc^©^ dose distribution is shown in Figure 1B. Radiotherapy was given using a Varian TrueBeam® linear accelerator with 6-MV photons under regular image guidance with cone-beam CT in 2 Gy per fraction (five fractions per week).

#### Proton Radiotherapy

Planning target volume for proton beam therapy was based on the CLEOPATRA protocol following image coregistration of axial T1 fat-saturated contrast-enhanced MRI scan outlining the contrast-enhanced volume as gross tumor volume (GTV protons) and adding a 5-mm clinical target volume (CTV) margin and an 8-mm PTV margin, respectively. Treatment planning was carried out on Siemens AG, Erlangen (Germany) Syngo treatment planning platform as inversely planned intensity-controlled (raster-scanned) protons using a two horizontal beams.

We prescribed 10 Gy (RBE) to the median of PTVprotons, which was encompassed by the 95% isodose level. Proton dose distribution is shown in Figure 1C. Radiotherapy was given at Marburg Ion Beam Therapy Center in Marburg, Germany, in 2 Gy (RBE) per fraction (five fractions per week) in active raster scanning under daily image guidance with orthogonal X-rays and position correction with a robotic treatment table in six degrees of freedom. Quantitative analyses of normal tissue effects in the clinic tolerance doses were adhered to for cumulative doses of photon and proton radiotherapy.

### Calculation of TTFields Intensity

In order to estimate field intensity distributions within the lesions, numerical simulations were performed using finite element method calculations and a realistic head model created as previously described (9, 10). Briefly, tumor tissues were segmented manually using a T1 contrast MRI of the patient. The region of the tumor was masked, and the resulting three-dimensional image was registered onto a realistic head model of a healthy individual, which serves as a deformable template. The registration process resulted in a non-rigid transformation mapping of the patient's head onto the deformable template. The inverse transformation was applied to the template to yield a model approximating the patient's head in the absence of the tumor. Finally, the tumor was placed back into the head, resulting in a realistic computational model of the patient's head. In order to establish that TTFields were delivered at therapeutic levels to the tumors, field intensities within a GTV and a proximal boundary zone (PBZ) were analyzed. The GTV comprised regions of enhancing tumor, and the PBZ comprised a zone 3 mm thick surrounding the tumor and resection cavity. The field was considered to deliver TTFields at therapeutic levels to the lesion if the median field intensity within the combined volume of the GTV and PBZ exceeded 1 V/cm.

## Discussion

This is an important report of a complete radiological response in a patient with GBM treated with photon therapy, proton boost, TMZ, and TTFields. Notably, this patient had unfavorable molecular markers and only had a surgical biopsy, not a surgical resection. While IDH mutations are more likely associated with secondary GBMs and are related to an increase in OS (11, 12), patients with MGMT promoter methylation have shown more favorable prognosis and prolonged survival (2). However, neither IDH mutation nor MGMT promoter methylation was detected (Figure 2). Further molecular analyses demonstrated a deletion of cyclin-dependent kinase inhibitor 2A (CDKN2A). In this patient, a CDKN2A deletion was detected, which is associated with shortened survival and limited response to radiation therapy in patients with GBM (13, 14). Resistance to TTFields therapy was assumed in a prior reported case with this constellation of characteristics (15), but TTFields resistance was not evident in this case study. However, the patient in this report has several positive outcome factors such as a younger age and a high KPS score. Additionally, a TERT promoter wild type (C228 and C250) was observed. A strong survival benefit for patients with TERT promoter wild type (C228 and C250) compared to patients with TERT promoter mutations is known (16). The negative match in the brain tumor methylation classifier is most likely due to intratumoral necrosis in the tissue. A no match in the brain tumor methylation classifier is not uncommon in recent studies (17). In a few cases, corticosteroid-induced regression of GBM is reported (18). The presented patient used corticosteroids only in the first treatment period until the end of radiation therapy. A direct influence of corticosteroids to the observed complete response (CR) seems rather unlikely. In rare cases, pilocytic astrocytoma could be misdiagnosed as GBM. In the histopathologic examination neither Rosenthal fibers nor eosinophilic granular bodies were found. In addition, in the molecular analysis, a strong signal for BRAF wild-type was observed, and no evidence for fusions of KIAA1549 and BRAF was detected.

### TTFields

**Figure 4:**
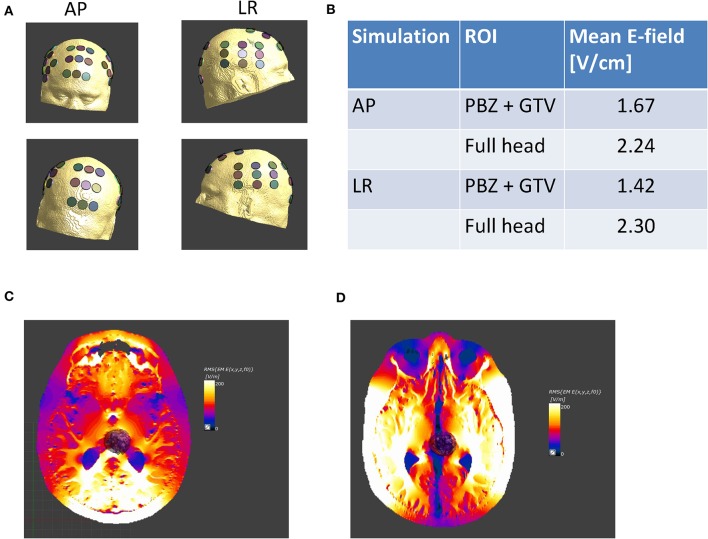
Tumor-treating fields intensity distribution. **(A)** Following model creation, virtual transducer arrays were placed on the patient model. The arrays were placed to match the array layout placed on the patient, as recorded in the clinical records, anterior-posterior (AP) and left-right (LR); **(B)** average field intensities within the GTV, comprising a region of enhancing tumor visible on the T1 contrast MRI at baseline and a PBZ 3 mm wide surrounding the tumor, were calculated. The table summarizes the average field intensity delivered by each pair of arrays to a tumor bed comprising the joint volume of the GTV and PBZ, as well as to the entire head. The table clearly shows that within the tumor bed, field intensities exceed the therapeutic threshold of 1 V/cm; electric field distribution shown for the AP **(C)** and LR **(D)** TTFields array configuration.

Despite the negative MGMT promoter methylation, the lack of IDH mutation, and CDKN2A deletion, the patient showed a complete radiological response 1 year after the completion of radiotherapy. Tumor-treating fields therapy was used together with maintenance TMZ for six TMZ cycles and was continued as a monotherapy for additional 14 months. The placement of the transducer arrays, through which TTFields are applied, was calculated using the NovoTAL™ methodology (9, 10). Despite the deep location of the lesion, numeric simulations (Figure 4) demonstrated that the field intensity delivered to the lesion was above therapeutic levels (>1 V/cm). These calculations support the efficacy of TTFields for deep lesions with a high morbidity risk for open resections. In addition, this patient showed a usage rate of TTFields therapy of 84% of the time greater than the recommended 75%, which is an independent prognostic factor for improved OS. This emphasizes the importance of both factors, a high field intensity at the lesion and high therapy compliance by the patient.

### Proton Boost Therapy

Currently, two randomized phase 2 trials have investigated the proton boost therapy and show it to be safe and feasible with reduced toxicity comparable to photon therapy (3). In one retrospective study with several limitations, OS was increased with proton therapy compared to photon therapy (19). The therapeutic advantage in treating GBM with proton therapy is the possibility to reduce or eliminate radiation exposure to non-tumor brain tissue. Currently, a randomized phase II trial studies how dose-escalated photon intensity-modulated radiation therapy or proton beam radiation therapy works compared with standard-dose radiation and chemotherapy in newly diagnosed GBM (NCT02179086). In relation to the current literature, it remains unclear whether there is a survival benefit for patients with GBM treated with proton therapy compared to photon therapy.

The patient in this report received proton boost therapy compared to the protocol of the CLEOPATRA trial (20). According to this protocol, a dose reduction of the radiation therapy in the non-tumor brain tissue was observed (Figure 1C).

### Evaluation of Treatment Response

The criteria of Response Assessment in Neuro-Oncology (RANO) published in 2010 are widely used to assess treatment response in first-line GBM therapy (21). The RANO criteria distinguish between progression, stable disease, partial response, and complete response. Based on the RANO criteria, a partial response was observed in this patient at 3 months, and a complete response was seen 1 year after the end of radiation therapy. Perfusion MRI could also be helpful in the assessment of treatment response in GBM (22). Glioma growth is often associated with changes in the blood–brain barrier, tumor angiogenesis, and higher local CBV and an increased CBF (23, 24). Eighteen months after completion of the radiation therapy, patient showed neither elevated CBV nor elevated CBF compared to the contralateral side (Figure 3). These radiological changes in the former tumor area support the diagnosis of a complete radiological response in the presented patient.

In summary, this case shows the combination treatment of chemoradiation with TMZ including proton boost, TMZ maintenance, and TTFields in a patient with pathologically confirmed GBM IDH wild type. The presented case shows that the combination of these therapies might prolong survival in GBM patients. However, either or both TTFields or proton boost may have contributed to this patient's good clinical outcome. Complete radiological responses were observed in other clinical trials, too (25). However, these results should be interpreted with caution and should not be generalized. For further evaluation, prospective data on the treatment of GBM patients by proton therapy in combination with TTFields are warranted for further recommendations.

## Conclusion

Despite negative predictors at the molecular level, complete radiological response was observed in a biopsied patient with pathologically confirmed GBM IDH wild type. This is the first report, to our knowledge, of a patient with a biopsied GBM IDH wild type receiving proton therapy followed by TTFields therapy.

## Data Availability Statement

The raw data supporting the conclusions of this article will be made available by the authors, without undue reservation, to any qualified researcher.

## Ethics Statement

Ethical review and approval was not required for the study on human participants in accordance with the local legislation and institutional requirements. The patients/participants provided their written informed consent to participate in this study. Written informed consent was obtained from the individual(s) for the publication of any potentially identifiable images or data included in this article.

## Author Contributions

MS identified the patient, reviewed the clinical history and the literature. MS and HD prepared the manuscript. HD and TA performed the genomic analyses and edited the manuscript. EU and MK contributed to the literature review and manuscript preparation. AJ and FE evaluated radiation dosing, contributed to the clinical history review, and reviewed the manuscript. BW reviewed the manuscript and the MRI scans.

## Conflict of Interest

MS has support for laboratory research and lectures from Novocure, Ltd. The remaining authors declare that the research was conducted in the absence of any commercial or financial relationships that could be construed as a potential conflict of interest.
